# Stable improvement in hand muscle strength in incomplete spinal cord injury patients by long-term paired associative stimulation—a case series study

**DOI:** 10.3389/fneur.2025.1486591

**Published:** 2025-02-04

**Authors:** Kirsi Holopainen, Aleksandra Tolmacheva, Ines Bersch, Piia Haakana, Markus Pohjonen, Erika Kirveskari, Jari Arokoski, Anastasia Shulga

**Affiliations:** ^1^BioMag Laboratory, HUS Diagnostic Centre, Helsinki University Hospital, University of Helsinki and Aalto University School of Science, Helsinki, Finland; ^2^International FES Centre^®^, Swiss Paraplegic Centre, Nottwil, Switzerland; ^3^Department of Physiology, University of Helsinki, Helsinki, Finland; ^4^Department of Internal Medicine and Rehabilitation, Division of Rehabilitation, Helsinki University Hospital and Helsinki University, Helsinki, Finland; ^5^HUS Medical Imaging Centre, Clinical Neurophysiology, Clinical Neurosciences, Helsinki University Hospital and University of Helsinki, Helsinki, Finland

**Keywords:** paired associative stimulation, transcranial magnetic stimulation, electrical stimulation, motor point integrity testing, spinal cord injury

## Abstract

**Introduction:**

Paired associative stimulation (PAS) consists of high-intensity transcranial magnetic stimulation and high-frequency electric stimulation of the peripheral nerve (high-PAS) and can induce plastic changes in spared corticospinal connections in individuals with spinal cord injury (SCI), leading to the restoration of motor function. The objective of this study was to investigate the long-term effect of high-PAS on hand function and muscle strength.

**Materials and methods:**

High-PAS was applied to four patients with chronic, incomplete, cervical-level SCI multiple times a week for as long as hand muscle strength improved. The median, ulnar, and radial nerves of one hand chosen by the patient were stimulated. Patients underwent Medical Research Council (MRC) manual muscle testing monthly during the stimulation period and were followed for 12 months after the stimulation.

**Results:**

Strength increased in both the stimulated and non-stimulated hands. In muscles innervated by stimulated nerves, strength increased on average by 24.5% from pre- to post-conditions (*p* = 0.013). The achieved strength level was maintained for a minimum of 6 months after completing the stimulations. Patients were also evaluated with motor point (MP) integrity testing to estimate the extent of lower motor neuron damage. High MP integrity testing scores (low extent of damage) correlated positively with good MRC outcomes of the stimulated hand after high-PAS (*r* = 0.52, *p* ≤ 0.001).

**Conclusion:**

High-PAS may improve muscle strength of both the stimulated and contralateral sides. Stable results were achieved when stimulation was delivered as long as MRC score improved progressively. The optimal duration of high-PAS treatment remains unknown.

**Clinical trial registration:**

clinicaltrials.gov, identifier NCT03045744.

## Introduction

Spinal cord injury (SCI) is a life-changing condition that causes motor, sensory, and autonomic dysfunctions that lead to physical impairment and disability (1). SCI is classified as incomplete when some sensory or motor functions (or both) are preserved in the sacral segments and below the injured level, or complete, when all motor and sensory functions distal to the injury site, including the sacral segments, are absent (2, 3). Spontaneous recovery is most notable within the first 6 to 9 months after the injury (4). With comprehensive physical and occupational therapy in rehabilitation, patients with incomplete SCI can recover functionally (5). After the first year, in the chronic phase of SCI, conventional therapy primarily aims to improve the previously acquired functions. However, improvement in arm and hand muscle strength and function is also possible with training (4, 6). For individuals with tetraplegia, restoration of hand functions suitable for everyday activities represents the most critical aspect of their health and wellbeing (1, 7). This highlights the need for innovative therapeutic approaches.

Paired associative stimulation (PAS), a non-invasive neuromodulation technique consisting of concurrent transcranial magnetic stimulation (TMS) and peripheral nerve stimulation (PNS), is a promising treatment method in SCI (8). The neural volleys induced by TMS and PNS stimuli can be set to coincide at the cortical (8, 9) or spinal level (10–12). In facilitatory spinal PAS, TMS-induced descending signals and PNS-induced ascending signals in the corticospinal tract are timed to coincide with the corticomotoneuronal synapses in the spinal cord (10). These repetitive interactions between ascending and descending signals can increase motor-evoked potential (MEP) amplitudes and improve motor output in a manner of long-term potentiation (LTP)-like plasticity (10, 13, 14). In patients with SCI, PAS can induce neuroplastic changes in spared corticospinal connections, leading to restored motor function (15).

To promote the use of PAS in SCI rehabilitation, we have developed a “high-PAS” protocol that combines high-intensity TMS (100% of stimulator output) and high-frequency PNS (trains of six 1-ms pulses delivered at 100 Hz) (16). Paired pulses are given at 0.2 Hz every 5 s. In other PAS protocols, TMS is often delivered with an intensity based on the resting motor threshold (RMT) and PNS with single pulses (15), although the use of high-frequency pulse trains has also been reported (17, 18). Previous studies have shown that a 100-Hz PNS pulse train is more effective than higher or lower frequencies in high-PAS, and a 0.2-Hz frequency for paired pulses is superior to 0.4 Hz (19, 20). The idea of using high-intensity TMS and high-frequency PNS trains is based on interactions produced by multiple synchronous anti- and orthodromic volleys that can affect a wide net of connections (16). This has been hypothesized to lead to an LTP-like effect at the spinal level because the interactions leading to LTP overcome those leading to long-term depression (LTD) when the conditions favor both (21). Higher stimulation frequencies may also broaden the time window favoring LTP by affecting postsynaptic depolarization (22). The high-PAS protocol specifically aims to induce plasticity at the spinal level of the corticospinal tract and activate motor nerves (12, 16), although sensorimotor cortical activity is also modified (23).

During our previous trials, over 20 patients with incomplete SCI with different injury severities, ages, and times since injury achieved increased muscle force, improved hand function, or restored walking ability after high-PAS (16). As the neural pathways rewire after SCI more efficiently with use and repetition (24), high-PAS has been delivered in long stimulation sequences and multiple sessions (16). PAS administered for 4–6 weeks has shown therapeutic potential in patients with chronic incomplete SCI (25, 26). One previous study also demonstrated a full recovery of hand muscle strength in manual muscle test scores and functional improvement after a 47-week high-PAS intervention in a patient with chronic incomplete SCI (27). This raised the need for a long-term high-PAS study with more patients.

Although the first high-PAS experiments with SCI patients have shown promising results, predicting the precise outcome of high-PAS is challenging. Motor point (MP) integrity testing detects upper and lower motor neuron lesions in patients with cervical SCI and tetraplegia (28). Usually, SCI results in an upper motor neuron lesion, with intact lower motor neuron and peripheral nerve function below the level of injury (28). Changes in peripheral nerve function can accompany SCI either acutely due to traumatic injury mechanisms in the anterior horn or chronically, for example, due to immobilization (5). The partial or complete lower motor neuron lesions occur usually at the level of the lesion and up to five levels below the centre of the lesion (28, 29). Differentiation of upper and lower motor neuron lesions improves predictability of the treatment of hand function and can affect rehabilitation, e.g., by the use of functional electrical stimulation or nerve transfers to reconstruct hand function (28). Since the effective use of PAS also requires at least partly intact peripheral innervation, MP integrity testing may be useful for predicting the outcome of PAS and for explaining individual differences.

In this study, we reported four patients of the five-patient study (clinicaltrials.gov, ID: NCT03045744) where high-PAS was administered to the upper extremities for as long as functional improvement was observed. The results for the first patient of this study (here patient 5) were reported by Rodionov et al. (27). In this patient, high-PAS was delivered to both hands, whereas in patients 1–4 (reported here), only one hand selected by the patient was stimulated. Patients 1–4 were followed up 12 months after the last stimulation session. We hypothesized that high-PAS would improve the muscle strength and manual dexterity of the stimulated hand regardless of the severity of incomplete SCI and time from injury. After the intervention, all patients were examined with MP integrity testing to determine whether the differences detected correlated with the high-PAS outcome.

## Materials and methods

### Patient recruitment

Four patients (three males, mean ± SD 52 ± 19 years) were recruited using the National Spinal Cord Injury Register. The inclusion criteria were cervical incomplete SCI (International Standards for Neurological Classification of Spinal Cord Injury and American Spinal Cord Injury Impairment Scale (AIS) B, C, D) (2) at the chronic stage (over 18 months) and an age range of 18 to 70 years. The exclusion criteria were concomitant brain injury, epilepsy, high intracranial pressure, metal implants in the head area, implemented electrical devices, and pregnancy. Patients continued their conventional rehabilitation program and medication during the study. Detailed information on patient characteristics is presented in Table 1. The Helsinki University Hospital Regional Committee on Medical Research Ethics approved the study. The entire study was performed in accordance with the Declaration of Helsinki. All participants provided written informed consent for participation.

**Table 1:** Patient characteristics.

Patient	Age, years	Sex	Time since injury	Etiology	AIS, NLI	Other therapies	Continuous CNS-active medication	Comorbid conditions
1	59	Male	4.5 years	Traumatic	D, C5	Physical therapy 40×60 min per year, occupational therapy 10×60 min per year.	Pregabalin 225 mg/day, tizanidine 24 mg/day, and baclofen 60 mg/day.	
2	66	Female	22 months	Traumatic	D, C4	Physical group therapy 1×60 min/ week.	Baclofen 10–20 mg/day and mirtazapine 7.5–15 mg/day.	Hypothyroidism, cervical spinal stenosis C3–C4, lumbar spinal stenosis L4–L5, and Alzheimer disease*.
3	24	Male	4 years	Traumatic	B, C7	Physical therapy 60×60 min per year, 10x pool therapy per year, and occupational therapy 20×60 min per year.	Baclofen 80 mg/day.	
4	59	Male	10 years	Non-traumatic	D, C4	Physical therapy 20×60 min per year.	Amitriptyline 30 mg/day.	Psoriasis and gastric bypass.

AIS, American Spinal Cord Injury Impairment Scale; NLI, neurological level of injury; CNS, central nervous system; *diagnosed during follow-up period after finishing stimulations.

### Study design

Patients selected the hand to be stimulated; all chose the dominant right hand. Patients were allowed to choose the stimulated hand to increase their motivation and commitment to the study to support their individual needs, and to enhance the possible positive effects on daily living. The contralateral hand was not stimulated. Based on earlier repetitive TMS studies (30) and previous high-PAS studies (16), PAS was administered 5 days a week for the first 2 weeks to maximize the induction of response to PAS and 3 times a week subsequently. One session included separate stimulations for three nerves (median [MN], ulnar [UN], and radial nerves [RN]), performed in alternating order. Patients were evaluated by an experienced physiotherapist before and once per month after starting high-PAS. Each patient was evaluated by the same physiotherapist, except for the last follow-ups of patient 2 and the pre-evaluation of patient 3. The physiotherapists were blinded to the setup, i.e., they did not know which side was stimulated. AT performed stimulation on patients 1–2, and KH performed stimulation on patients 3–4.

The primary outcome measure was the Medical Research Council (MRC) manual muscle test. The secondary outcomes were functional tests on hand dexterity (Box and Block Test and Nine-Hole Peg Test), hand and finger dynamometry scores, American Spinal Injury Association (ASIA) sensory and motor scores, Modified Ashworth Scale (MAS) spasticity score, International Spinal Cord Injury Pain Basic Data Set (ISCIPBDS), World Health Organization Quality-of-Life (WHOQOL) assessment, and the Spinal Cord Independence Measure (SCIM). High-PAS sessions continued as long as the MRC score level or other outcomes (or both) improved. If the MRC score decreased or did not increase further from one evaluation to another, stimulations were terminated. However, secondary outcomes and patient opinion were also considered. Stimulation timelines of each patient are summarized in Table 2. Follow-up was performed at 1 month, 3 months, 6 months, and 12 months after the last high-PAS session.

**Table 2:** Stimulation parameters.

Patient	Stimulated hand	Duration of PAS	PNS intensity (mA)	F latency (ms)	MEP latency (ms)	ISI (ms)
1	Right	25 weeks				
Median			6	29.8	26.8	+3
Ulnar			6	31.3	32	−1
Radial			10	23.5	21.1	+2
2	Right	14 weeks				
Median			5	29.9	27.6	+2
Ulnar			11.4	31.8	25.2	+7
Radial			10	11.3	17	−6
3	Right	16 weeks				
Median			15–19 (est.)	–	27.3	+0.5 (est.)
Ulnar			15	27.3	26.87	+0.5
Radial			15	18	16.57	+1.5
4	Right	19 weeks				
Median			4	27.8	24.83	3
Ulnar			9.5	26.8	24.17	2.5
Radial			19	18.4	16.26	2

PNS, peripheral nerve stimulation; MEP, motor-evoked potential; ISI, interstimulus interval; + in ISI, PNS before TMS; − in ISI, PNS after TMS.

### High-PAS settings

TMS was performed using a figure-of-8 coil of an eXimia magnetic stimulator with an MRI-based navigation system (Nexstim Ltd., Helsinki, Finland). Cortical representations of the abductor digiti minimi (ADM), abductor pollicis brevis (APB), and extensor digitorum communis (EDC) muscles were mapped. The site where the TMS elicited the largest MEPs in the muscle innervated by the nerve to be stimulated was chosen as the TMS “hotspot” for each muscle–nerve pair individually. All three hotspots were stored in the TMS device, and the same hotspots were used throughout the stimulations. Thereafter, the resting motor threshold (RMT) of the muscles was identified as a minimum TMS intensity eliciting at least 50 μV in 5/10 stimuli (31). For interstimulus interval (ISI) calculation, MEP latency was measured from an average of 15 MEPs elicited by TMS of 100% stimulator output.

**Figure 1:**
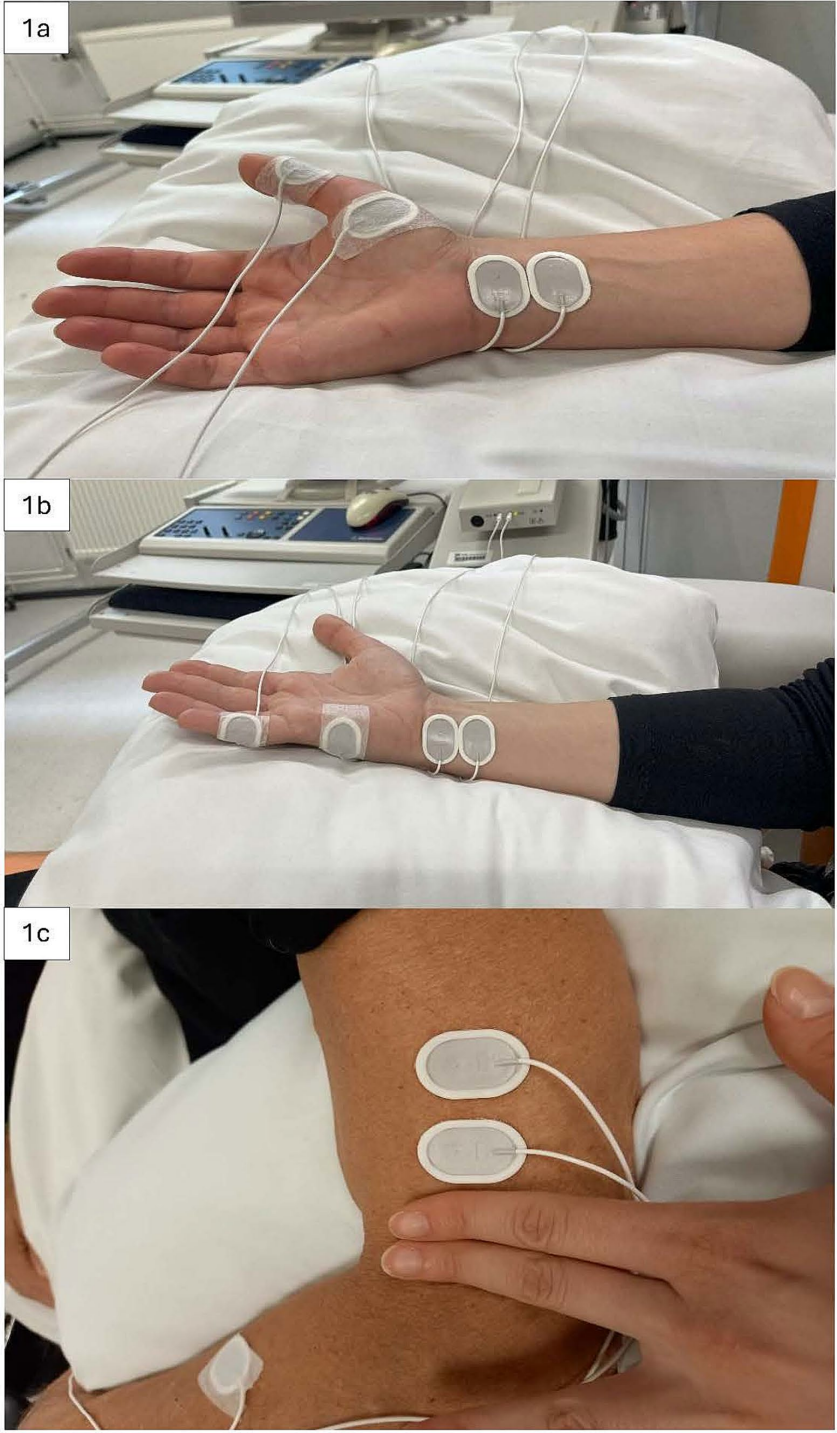
**(A–C)** Electrode placement of stimulating electrodes used for the F-response measurement and PNS in high-PAS. **(A)** Median nerve; **(B)** Ulnar nerve; **(C)** Radial nerve.

The MN, UN, and RN were stimulated. Self-adhering surface electrodes (Neuroline 720, AMBU A/S, Ballerup, Denmark) were used for electrical stimulation and F-response measurement. Two stimulation electrodes were placed in the middle of the inner wrist for MN, in Guyon’s canal for UN, and laterally above the elbow for RN (32). During RN stimulation, electrodes were gently pressed against the humerus to enable effective nerve activation. The same electrode montage was used for the F-response measurement and PNS in high-PAS (Figure 1). Two recording electrodes were placed at a belly–tendon montage over APB, ADM, and EDC during MN, UN, and RN F-response measurements, respectively.

PNS was delivered, and F-responses were measured using a Keypoint device (Natus Medical Inc., Pleasanton, CA, United States). An experienced clinical neurophysiologist measured F-responses at supramaximal intensity with a 0.2-ms pulse for determining minimum latency out of 10 responses (33), which was used for ISI calculation. Thereafter, minimum intensity-evoking F-responses [1 F-response out of 10 stimuli (34)] were determined with a 1-ms pulse stimulation, and this intensity and pulse length were used for PNS in high-PAS. F-responses from MN were not detected in patient 3. ISI and PNS onset intensity were set to be the same as for UN (15 mA). After 2 months of PAS, the intensity was gradually increased to 19 mA, which was still tolerated by the patient.

ISI between TMS and PNS was set to synchronize arrivals of a single TMS pulse and the first pulse of the PNS train in the corticomotoneuronal synapses in the spinal cord (10). This was calculated individually for each patient by the formula [F latency – MEP latency] (see Shulga et al. (35) for a detailed description).

During high-PAS, a single TMS pulse was delivered at 100% of the maximum stimulator output (MSO). PNS was delivered as a 100-Hz train consisting of six 1-ms square wave pulses. Two hundred forty PAS sequences of one target muscle were triggered through Presentation^®^ software (Neurobehavioral Systems Inc., Albany, United States) at 0.2 Hz. The session duration for one nerve was 20 min. Patients voluntarily activated the target muscles with each PAS (little finger abduction/flexion of IV and V fingers for ADM, abduction and flexion at the carpometacarpal joint of the thumb for APB, and wrist and finger extension for EDC). The patients were advised to slightly contract the muscle just before the TMS pulse to draw their attention to the stimulated hand and decrease motor thresholds (16). The three target nerves were stimulated in alternate order during each PAS session. Details on the stimulation parameters of the patients are presented in Table 2. Due to occasional initial discomfort, PNS intensity was gradually adjusted to the target level during the first 10–15 pulses of stimulation in the first stimulation sessions. Habituation to the PNS intensity occurred during the first stimulation sessions. All patients tolerated 100% MSO TMS intensity without adverse events. During hand representation area stimulation, slight face sensations, twitches, or involuntary movements of the arm might have occurred.

### Outcome measures

MRC, ASIA motor and sensory scores, hand dynamometry, hand dexterity tests, pain score, spasticity score, and SCIM were evaluated at each evaluation point (baseline and pre, end of PAS, and 1-month, 3-month, 6-month, and 12-month follow-ups). Both hands were assessed. WHOQOL assessment was performed before, immediately after long-term PAS, and at 12 months of the follow-up. To detect intra-individual variability in the tests, two baseline assessments were performed before PAS administration, separated by a minimum of 1 month and a maximum of 7 months. These evaluations were performed on days when the patients had their usual general health to preclude confounding factors. Patient 1 had the evaluation after 5 months of stimulation postponed for a few weeks due to elevated blood pressure and related unwellness. The rescheduled evaluation was used as the “End of PAS” as no further improvement was detected in MRC, and stimulations were discontinued after this evaluation. Patient 1 also missed the 12-month follow-up because of temporary health deterioration unrelated to this study.

Voluntary muscular strength was assessed for each muscle separately with the MRC scale as follows: 0 = no contraction, 1 = flicker or trace of contraction, 2 = active movement, with gravity eliminated, 3 = active movement against gravity, 4 = active movement against gravity and resistance, and 5 = normal power (36). The scapula and shoulder muscles were tested in addition to the muscles innervated by MN, UN, and RN and analyzed separately.

Strength in the key hand muscles (C2–C8, T1) and sensory score in hands (C2–C8, T1–T2) were assessed according to the ASIA examination sheet (2). The sensory test consisted of light touch (LT) and pin prick (PP) tests. Grip strength was measured using an Exacta™ Hydraulic Hand Dynamometer (North Coast Medline, Inc., United States). The dynamometer was adjusted to the size of the patient’s hand. Pinch dynamometry was performed using a Baseline^®^ Mechanical Pinch Gauge (Fabrication Enterprises Inc., United States) for key pinch, tip pinch, and palmar pinch. The patient had three attempts for each test, and the best result in kg was used for analysis (37).

Unilateral gross manual dexterity for both hands was evaluated using the Box and Block Test (BBT) (38). The result reflects the number of transferred blocks in 1 min. Unilateral finger dexterity was measured using the Nine-Hole Peg Test (9-HPT) (39). The result was expressed as the number of successful placements performed in 50 s. Hand spasticity was assessed using the Modified Ashworth Scale (MAS) (40). MAS scores range from 0 to 4, where 0 represents normal muscle tone and higher scores represent spasticity.

SCIM is a self-reported evaluation of daily activity divided into the following three domains: self-care, respiration and sphincter management, and mobility (41). SCIM scores range from 0 to 100, with higher scores indicating better daily performance. Pain was measured using the ISCIPBDS (42). The overall ISCIPBDS score (range 0 to 36 points, higher scores indicate worse pain) was used in this study. The WHOQOL-BREF is a quality-of-life assessment divided into four domains, namely, physical, psychological, social, and environmental domains (43). The total raw score of all domains was used; a higher score indicates a better quality of life.

Subjective reports and verbal feedback were collected throughout the study. The patients were asked to verbally describe changes they experienced in their hand functions in daily life. Adverse events and other (not study-related) events causing changes in patients’ status and wellbeing were also collected.

### Motor point integrity test

A motor point (MP) integrity test of forearm muscles with electrical stimulation was conducted as described in Bersch et al. (28). MP was defined as the site that produces a maximal selective and defined muscle response at the lowest stimulation intensity (44). Five muscles innervated by the radial nerve (extensor carpi ulnaris [ECU], extensor carpi radialis [ECR], extensor digitorum communis [EDC], extensor pollicis longus [EPL], and abductor pollicis longus [APL]) on the dorsal aspect of the forearm and three muscles innervated by the median nerve (pronator teres [PRT], flexor digitorum profundus [FDP], and flexor pollicis longus [FPL]) on the palmar aspect of the forearm were tested using a Mettler 212 Digi-Tens (Mettler Electronics Corp., United States) electrical stimulator. One self-adhesive electrode (Everyway Medical Instruments Co., Ltd., Taiwan) was attached to the epicondyle as a reference electrode. A pen electrode with a diameter of 0.8 cm (ZMI Electronics Ltd., Taiwan) was used for stimulation. The MPs of selected muscles were detected with a pen electrode using a cartography system and calculated stimulation points (28) (Supplementary file). In addition, palmar interossei I, II, and III (IOD1-3) muscles were tested. The IOD1 MP was defined as the midpoint of the first and second metacarpophalangeal joints of the thumb (45). IOD2 MP was searched manually from the base between the index finger and middle finger and IOD3 MP between the middle finger and ring finger. The test pulse width was 260 μs with a frequency of 35 Hz. The contraction was elicited in the muscles by stimulation intensity between 20 and 50 mA. The results were categorized into innervated, partially denervated, and denervated according to muscle response (28).

### Statistical analysis

MRC was pre-registered at clincaltrials.com as the primary outcome measure at timepoints before PAS, end of PAS, and 1-month follow-up. MRC results were analyzed using IBM SPSS Statistics 28.0. Non-parametric tests were used as the data were non-normally distributed in the Kolmogorov–Smirnov and Shapiro–Wilk tests. MRC data from three timepoints (pre, end of PAS, and 1-month follow-up) were analyzed using Friedman’s two-way ANOVA for related samples. A *post-hoc* test was performed using the Dunn–Bonferroni test. The alpha level was set at 0.05/3 = 0.0167 (Bonferroni correction) to control multiple comparisons. The results from the right (stimulated) hand and left (unstimulated) hand were analyzed separately (*n* = 4 hands). The same statistical analysis was also performed for MRC results from all timepoints (pre, end of PAS, and 1-, 3-, 6-, and 12-month follow-ups); the alpha level was set at 0.05/15 = 0.0033 and without results from 12-month follow up; the alpha level was set at 0.05/10 = 0.005. The mean of baseline and pre-measurements was calculated to control day-to-day variation. Muscles with a maximum score of 5 in pre-evaluation were removed from the analysis as it was not possible to detect further improvement in these muscles with MRC. The mean MRC score of all included muscles was calculated for each evaluation, and these values were used for analysis. In addition, changes between pre and other evaluations were calculated. The correlation between MRC changes from pre to end of PAS and MP integrity test was calculated using Pearson’s product–moment correlation. All muscles with a maximum score pre-evaluated to 5 were also removed from this analysis. Due to the small number of patients, the results from other measurements that were not pre-registered at clincaltrials.com are presented using descriptive methods. All data are presented as mean ± standard error.

## Results

### Primary outcome

MRC scores did not differ between baseline and pre-evaluations (*p* = 1.00; Wilcoxon signed-rank test in both hands). The mean of these measurements was calculated and used for analysis; the timepoint “Pre” later in the text refers to this value. MRC scores of stimulated muscles differed significantly between the three timepoints (pre, end of PAS, and 1-month follow-up) in Friedman’s test in stimulated (*n* = 4) [X^2^(2)=6.5, *p* = 0.039] and unstimulated hands (*n* = 4) [X^2^(2)=6.5, *p* = 0.039]. The *post-hoc* test indicated that MRC scores increased significantly from pre to end of PAS (stimulated +0.77 ± 0.1 points, *p* = 0.013, unstimulated +0.71 ± 0.14 points, *p* = 0.013; Dunn–Bonferroni test) but not from pre to 1-month follow-up (stimulated +0.73 ± 0.14 points, *p* = 0.077, unstimulated +0.63 ± 0.07 points, *p* = 0.077). MRCs between the end of PAS and 1-month follow-up did not differ (*p* = 0.480).

### Additional timepoints

**Figure 2:**
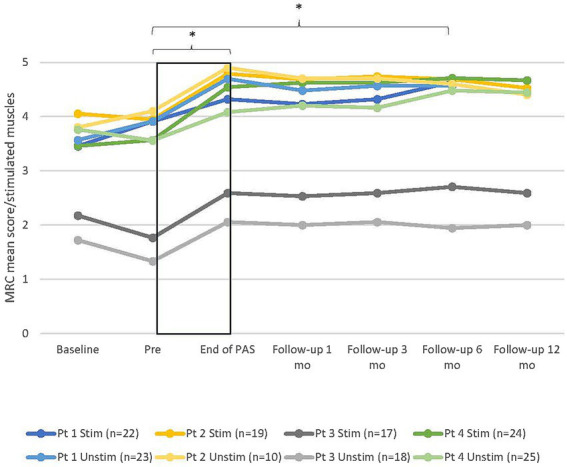
Individual development of manual muscle testing (MRC) mean score of muscles innervated by the median, ulnar, and radial nerves in all patients in both hands. Stim, stimulated (right); Unstim, unstimulated (left); *n*, number of included muscles; 0, no contraction; 5, maximum score; Pt, patient; Baseline, additional evaluation 1–7 months before pre. * = statistical difference between two measuring points. Rectangular black box = stimulation time.

MRC scores between all six timepoints (pre, end of PAS, and 1-, 3-, 6-, and 12-month follow-ups) did not differ significantly in the stimulated [X^2^(5)=9.515, *p* = 0.090] or unstimulated hand [X^2^(5)=9.515, *p* = 0.090]. This result was affected by the dropout of one patient after 6 months of follow-up. When the 12-month follow-up was excluded from the analysis and all four patients included, Friedman’s test revealed a significant difference between the timepoints in the stimulated [X^2^(4)=12.101, *p* = 0.017] but not in the unstimulated hand [X^2^(4)=9.455, *p* = 0.051]. The *post-hoc* test showed a significant difference between the pre and 6-month follow-up in the stimulated hand (+0.9 ± 0.1 points, *p* = 0.002; Dunn–Bonferroni test) and almost a significant difference between the pre and 3-month follow-up (+0.78 ± 0.1, *p* = 0.01). MRC scores did not differ between the other timepoints. The individual development of MRC mean scores (Figure 2) showed that despite the lack of statistical significance at every timepoint, the MRC mean scores remained above the pre-level throughout the follow-up time up to 12 months in all three patients.

MRC scores of non-stimulated scapula and shoulder area muscles increased from before to End of PAS, especially in patients who had strength deficiencies in these muscles. Scores for patient 1 increased from 4.2/4.4 to 5 and for patient 4 from 3.6 to 4.9 in the right hand and from 3.5 to 5 in the left hand. Patient 2 had maximum test scores for the right arm and patient 3 for both arms already in the pre-test, so detecting further improvement was not possible.

**Figure 3:**
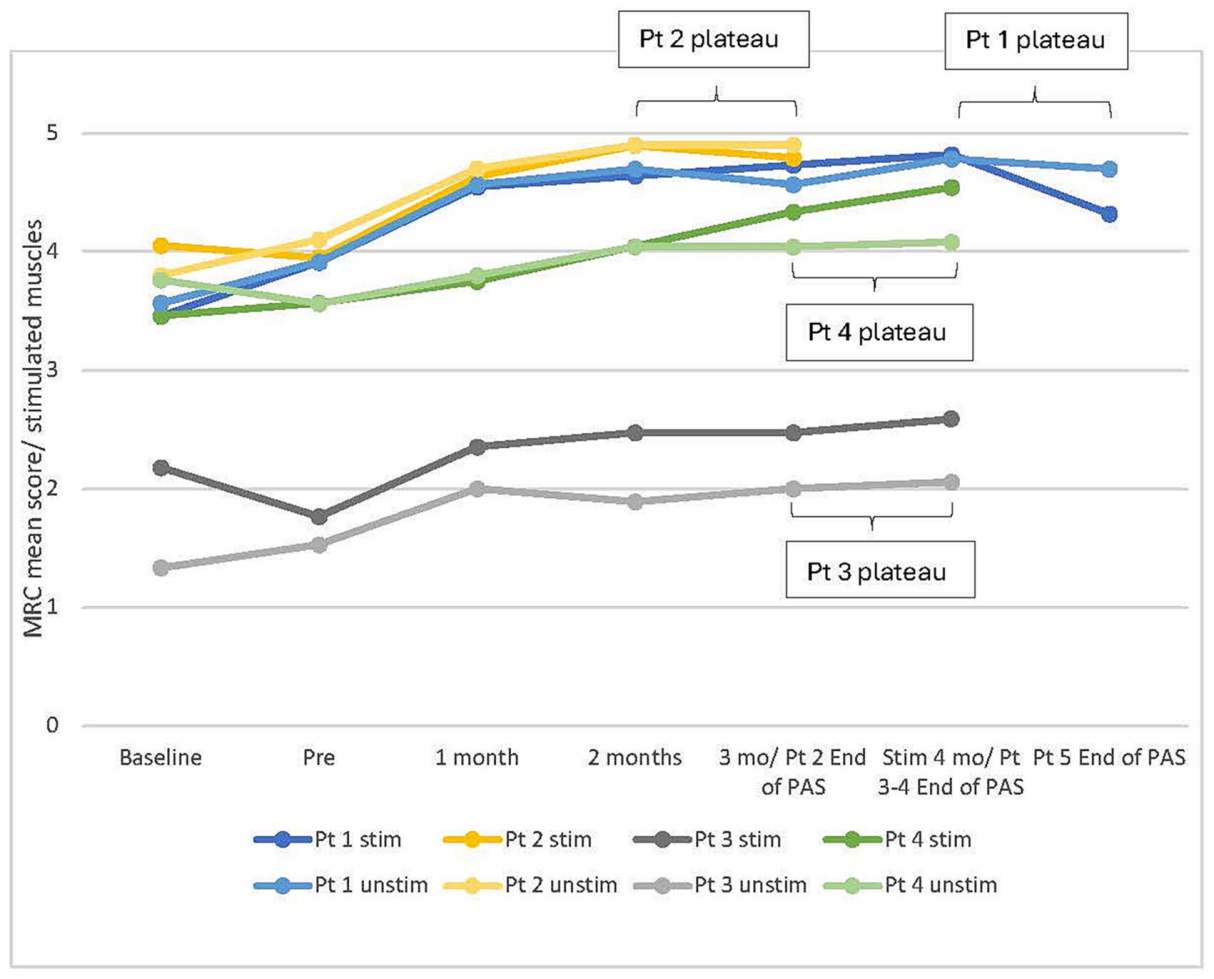
Individual development of manual muscle testing (MRC) mean score of muscles innervated by the median, ulnar, and radial nerves in all patients in both hands during stimulation period. Stim, stimulated (right); Unstim, unstimulated (left); *n*, number of included muscles; 0, no contraction; 5, maximum score; Pt, patient; Baseline, additional evaluation 1–7 months before pre.

The duration of PAS required to achieve the plateau of MRC varied between patients (range 14–25 weeks, mean 18.5 ± 2.4 weeks). The development of MRC scores for each patient and hand during the stimulation period, including the most notable phases of increase and plateau, is presented in Figure 3.

### Secondary outcomes

**Figure 4:**
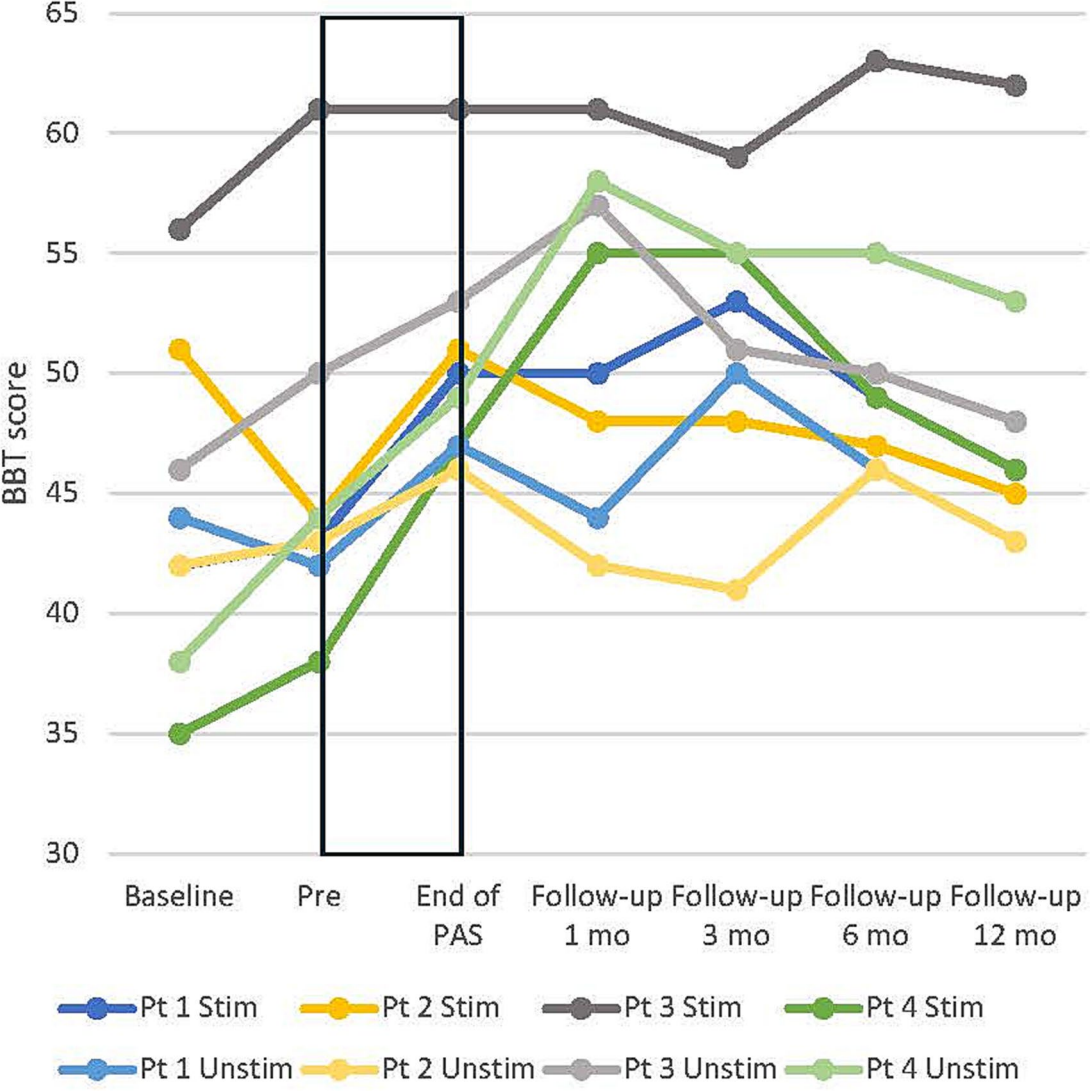
Individual development of box and block test (BBT) score in all patients in both hands. Stim, stimulated (right); Unstim, unstimulated (left); Pt, patient; Baseline, additional evaluation 1–7 months before pre. Rectangular black box = stimulation time.

From pre to end of PAS, BBT scores increased in all patients on average by 6 ± 1.8 points (15%) in the stimulated hand and 5.1 ± 1 points (12%) in the unstimulated hand. BBT scores remained above the pre-level up to the 12-month follow-up (Figure 4). 9-HPT scores of the stimulated hand did not increase from the pre-test to the end of PAS; three of four patients had between 17 and 18 points already in the pre-test (18 points is the maximum). The unstimulated hand 9-HPT score decreased slightly from pre to end of PAS, and the results varied between different evaluations. Group-level results are presented in Table 3.

**Table 3:** Secondary outcomes (mean ± standard error).

Test	Baseline	Pre	End of PAS	1-month follow-up	3–month follow-up	6-month follow-up	12-month follow-up
9-HPT STIM (pcs)	14.8 ± 2.6	14.8 ± 2.9	14.8 ± 2.4	17 ± 1	18 ± 0	17 ± 1	14.7 ± 3.3
9-HPT UNSTIM (pcs)	15.8 ± 2.3	14.5 ± 2.1	11.5 ± 2.8	16.3 ± 1.8	13.8 ± 1.9	13.8 ± 2.7	10 ± 4
Hand dynamometry STIM (kg)	20 ± 5.3	21.7 ± 3.8	26.3 ± 3.8	28.7 ± 4.7	26 ± 4	26 ± 4.8	22 ± 4.1
Hand dynamometry UNSTIM (kg)	22.3 ± 1.2	22 ± 3.6	27 ± 4	25 ± 2.9	27.7 ± 5	27.3 ± 4	21.5 ± 0.4
Tip pinch STIM (kg)	4.2 ± 1.5	4 ± 1.3	3.9 ± 1.4	5 ± 1.7	3.9 ± 1.4	4.6 ± 1.7	3.8 ± 2
Tip pinch UNSTIM (kg)	3.4 ± 1.2	3.8 ± 1.3	4.6 ± 1.7	4.9 ± 1.7	4.4 ± 1.8	4.6 ± 1.6	3.9 ± 2
Palmar pinch STIM (kg)	5.2 ± 1.8	4.1 ± 1.4	5.2 ± 2	5.8 ± 2.1	5.5 ± 2.2	5.3 ± 2.2	3.8 ± 2.1
Palmar pinch UNSTIM (kg)	5.5 ± 1.8	4.2 ± 1.4	5.7 ± 2.1	6.2 ± 2.2	5.4 ± 2.1	5.4 ± 2	4.1 ± 2.2
Key pinch STIM (kg)	4.75 ± 1.7	5.6 ± 1.9	5.8 ± 1.9	6.4 ± 2.1	6.4 ± 2.2	6.3 ± 2.2	4.8 ± 2.2
Key pinch UNSTIM (kg)	4.2 ± 1.4	5.9 ± 2	5.6 ± 2	6.2 ± 2.3	5.6 ± 2	5.9 ± 1.9	4.3 ± 2.1
ASIA motor STIM (points)	21.5 ± 1.6	21 ± 1.8	24.0 ± 0.7	23.8 ± 0.6	24.0 ± 0.7	24.0 ± 0.7	23.0 ± 0.6
ASIA motor UNSTIM (points)	22.3 ± 1.5	20.8 ± 1.8	21.8 ± 2.1	21.8 ± 2.1	21.8 ± 2.1	22.3 ± 2.1	21.0 ± 2.6
ASIA LT STIM (points)	14.5 ± 1.7	15.3 ± 1	16.8 ± 0.5	15.5 ± 0.3	16.0 ± 0.7	16.3 ± 0.6	15.7 ± 0.3
ASIA LT UNSTIM (points)	14.8 ± 0.9	15.5 ± 1	16.3 ± 0.3	16.0 ± 0.4	16.0 ± 0.9	16.3 ± 0.6	16.3 ± 0.9
ASIA PP STIM (points)	11.8 ± 1.7	11.5 ± 2.5	14.8 ± 1.4	14.3 ± 1.3	14.3 ± 1.2	13.0 ± 2.2	12.7 ± 1.1
ASIA PP UNSTIM (points)	14.3 ± 2.3	13.5 ± 2	16.3 ± 1.0	14.0 ± 1.5	15.5 ± 1.0	15.3 ± 1.6	12.7 ± 0.9
MAS STIM (points)	2.8 ± 1	3.8 ± 1.3	2.0 ± 0.9	2.8 ± 1.1	2.0 ± 1.1	3.5 ± 1.3	3.0 ± 2.1
MAS UNSTIM (points)	2.1 ± 1.3	1.3 ± 0.6	0.8 ± 0.5	1.0 ± 0.4	1.0 ± 0.4	1.5 ± 0.9	1.3 ± 0.3
ISCIPBDS (points)	11.25 ± 5	6.8 ± 6.1	7.8 ± 5.7	12.3 ± 7.1	13.0 ± 5.1	6.0 ± 4.8	11.0 ± 6.4
WHOQOL (points)		88.5 ± 6.3	85 ± 9.9				95.3 ± 9.7

STIM, stimulated (right); UNSTIM, unstimulated (left); 9-HPT, Nine-Hole Peg Test; ASIA, American Spinal Injury Association; LT, light touch; PP, pin prick; MAS, Modified Ashworth Scale; ISCIPBDS, International Spinal Cord Injury Pain Basic Data Set; WHOQOL, World Health Organization Quality of Life.

Hand dynamometry and palmar pinch scores increased slightly from pre to end of PAS and remained increased up to the 6-month follow-up. Key pinch increased slightly in the stimulated hand and tip pinch in the unstimulated hand. Patient 3 was unable to execute hand or pinch dynamometry in pre-evaluation but had a slight improvement in the key pinch and palmar pinch during the intervention. Group-level results are presented in Table 3.

**Figure 5:**
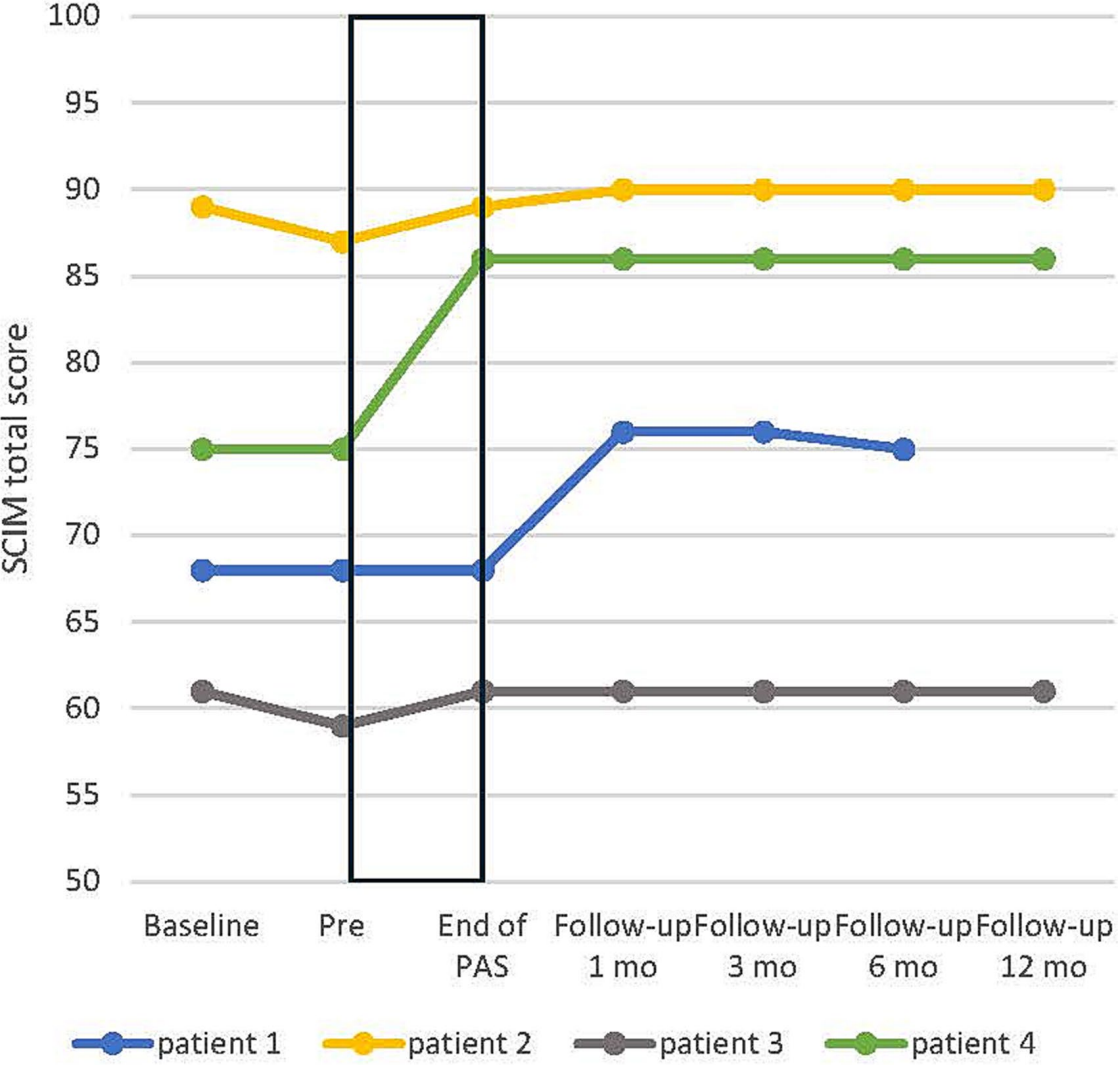
Individual development of spinal cord independence measure (SCIM) total score in all patients. Baseline, additional evaluation 1–7 months before pre. Rectangular black box = stimulation time.

SCIM’s overall score increased on average by 3.75 ± 2.46 points from pre to end of PAS. The change in overall score during intervention and during follow-up is presented in Figure 5.

ASIA motor score increased by 3 ± 1.4 points from pre to end of PAS in the stimulated but not in the unstimulated hand. ASIA sensory scores did not show notable change; the stimulated hand PP score increased by 3.3 ± 1.9 points from pre to end of PAS, but the increase was 1.5 ± 1.7 points after 6 months. Spasticity did not change according to the MAS score in the stimulated or the unstimulated hand. Group-level test results are presented in Table 3.

According to ISCIPBDS, neuropathic pain did not change after high-PAS (Table 3). One of four patients had continuous pain (ISCIPBDS score over 20/36 points in every evaluation), one patient did not have pain at all, and pain scores varied throughout the intervention for two patients. The mean WHOQOL total score decreased slightly from pre to end of PAS but increased from pre to 12-month follow-up (Table 3). In WHOQOL sub-questions related to quality of life and the ability to perform daily activities, quality of life increased from 3.75 ± 0.25 to 4.3 ± 0.3 and the ability to perform daily activities from 3.75 ± 0.25 to 4 from pre to end of PAS.

### Other results

MP integrity test results, including data from patient 5 from Rodionov et al. (27), are presented in the Supplementary file. The MP integrity test revealed that patients 1 and 4 had only fully innervated muscles and patients 2 and 5 had individual completely or partially denervated muscles in the stimulated right hand. Patient 3 had partial or complete denervation in all flexor/ palmar side muscles, including interossei 1–3 in his right hand. In the left hand, patients 1 and 3 had individual completely or partially denervated muscles. MP integrity test results were combined with MRC score changes (from pre to end of PAS) of corresponding muscles. Thirty-eight right-hand muscles and 39 left-hand muscles from all patients were analyzed. MRC and MP integrity test results correlated positively (*r* = 0.515, *p* ≤ 0.001) in the right-stimulated hand but not in the left-unstimulated hand (0.267, *p* = 0.101; Pearson’s correlation coefficient).

Subjective reports on the long-term effects of high-PAS were collected from patients throughout the study. The most common improvement was that the stimulated hand felt stronger and more flexible. Examples of improved finger movements from patients 1 and 4 can be seen in Supplementary Videos. Patients also reported a tighter grip and improvements in their handwriting, ability to grasp objects, and ability to make a fist more easily. No adverse events were reported during the study.

## Discussion

High-PAS increased muscle strength in MRC in both the stimulated and contralateral upper extremities when administered for as long as improvement was detected. This improvement persisted in the stimulated hand for 6 months after the last stimulation session in all patients and for up to 12 months in the three patients who participated in the last follow-up. Some improvements in hand dexterity (BBT), grip strength, and SCIM were also observed. Spasticity, finger dexterity (9-HPT), pain, and sensory function remained unchanged. These outcomes are consistent with previous high-PAS studies (16). Increased muscle strength and hand function are probably induced by plasticity at corticomotoneuronal synapses of the spinal cord. Strengthening of the synapses may cause more effective recruitment and faster firing of motor neurons, and these changes are linked to increased contraction force and motor learning (10).

Changes observed in this study were less dramatic than those described by Rodionov et al. (27) in an analogous experiment that stimulated both hands. This may indicate that stimulation of one hand is less effective than bilateral stimulation. Stimulation of both hands may increase the probability of reaching more intact circuits in the corticospinal tract and thus promote more rerouting of projections, strengthening of synapses, or both (16). Improvement in the unstimulated hand after high-PAS was also reported by Tolmacheva et al. (26). Enhanced functionality of the stimulated hand may motivate patients to participate in tasks using both hands in a more adaptable manner, facilitating the recovery of both hands. High-PAS may also induce cross-activation, a bilateral increase in corticospinal excitability reported after unilateral strength training (46), motor task performance (47), and electrical stimulation (48). Kolzenburg et al. described a similar contralateral change and suggested that either overlapping individual dendrites of motor neurons or commissural interneurons in the spinal cord could transfer the activation to the contralateral side and also modify the inhibition–excitation balance (49).

All reported improvements were preserved for 6–12 months after the end of stimulations. Although this was the first high-PAS study where the follow-up time was longer than 6 months, one patient did not participate in the last follow-up due to health deterioration unrelated to this study. This dropout affected group-level results of the last timepoint. Typically, the test results started to improve after the first month of active stimulation (Figure 3), improved over an average of 18 weeks, and remained at a relatively similar level during the follow-up. The stimulation period exceeded 20 weeks only for patient 1, and his results plateaued during the last month of stimulation. Nevertheless, the optimal duration of the stimulation period appears to be individual because all patients responded to the stimulation differently. For example, patient 4 reached the peak of increase in MRC later than other patients in this study, and in the case reported by Rodionov et al. (27), improvement continued until 47 weeks of stimulation. It cannot be confirmed whether another strategy for determining when stimulation should be discontinued would have been more successful and what would have been the optimal stimulation time for each patient. Continued improvement was observed after the end of the stimulation in some cases but rarely longer than 1 month.

Patients 1 and 4 benefited more from high-PAS than patients 2 and 3. Our previous results suggest that younger patients with less severe and more recent injuries derive more benefit from high-PAS and more rapidly than others (16). Patient 3 was younger but had more severe injury and a more prominent lower motor neuron lesion as detected by MP integrity test than the other patients. MP integrity test results correlated positively with pre-end of PAS changes in MRC in the stimulated hand, suggesting that fully innervated muscles improved more through high-PAS than completely or partially denervated muscles. A small number of denervated muscles might have affected the result of the contralateral hand. Patients who responded better in this study and in the previous study by Rodionov et al. (27) had different lesion severities, ages, and times from injury, although no patients had signs of lower motor neuron lesions. Thus, differentiation of upper and lower motor neuron lesions with the MP integrity test may assist in predicting the outcome of high-PAS, and it deserves further investigation. Although patient 2 had a mild and relatively recent injury, she was older and was diagnosed with Alzheimer’s disease during the follow-up period after finishing the stimulation sessions. The diagnosis of Alzheimer’s disease was not known at the time of recruitment, and this disease probably affected hand and pinch dynamometry, which did not improve (50).

The limitations of this study are the small number of participants and the lack of a control group or sham condition. Each patient acted as their own control, and a stable condition before intervention was confirmed by two baseline evaluations. All SCIs were chronic (>1.5 years since injury). Patients neither changed their physical therapy or training routine or medication nor received other new treatments during the intervention. Thus, it is likely that the achieved improvements in muscle strength and hand function were due to high-PAS. However, learning effects are possible in all behavioral and functional tests (which are repeated several times), and we cannot completely distinguish these effects from those caused by the stimulation. Instead of comprehensive statistical analysis, this study focused on exploring various aspects of functional ability at the individual and group levels and the permanence of gained abilities.

## Conclusion

An individually designed high-PAS intervention where stimulation is applied for as long as improvement can be detected (on average 18 weeks) is an attractive option for improving especially hand muscle strength in the long term in patients with chronic, incomplete, cervical SCI. MP integrity testing may help in identifying patients who are likely to achieve beneficial outcomes after high-PAS.

## Data Availability

The raw data supporting the conclusions of this article will be made available by the authors, without undue reservation.
