# Supplementary material for: Stable improvement in hand muscle strength in incomplete spinal cord injury patients by long-term paired associative stimulation—a case series study
Source: Front Neurol. 2025 Feb 4;16:1486591. doi: 10.3389/fneur.2025.1486591 (PMC11832407; doi:10.3389/fneur.2025.1486591)
Supplement: Supplementary file 1 [file Data_Sheet_1.pdf]

Supplementary file

Supplementary file 1 Motor point integrity test results

RIGHT	AIS, NLI	ECU	ECR	EDC	EPL	APL	PRT	FDP	FPL	IOD 1	IOD 2	IOD 3
pt1	D, C5	2	2	2	2	2	2	2	2	2	2	2
pt2	D, C4	2	0	2	2	2	2	2	2	2	2	2
pt3	B, C7	2	2	1	2	2	2	1	0	1	0	0
pt4	D, C4	2	2	2	2	2	2	2	2	2	2	2
pt5	B, C7	1	2	2	2	2	2	2	2	1	2	2
LEFT	AIS, NLI	ECU	ECR	EDC	EPL	APL	PRT	FDP	FPL	IOD 1	IOD 2	IOD 3
pt1	D, C5	2	2	1	2	2	2	1	2	2	2	2
pt2	D, C4	2	2	2	2	2	2	2	2	2	2	2
pt3	B, C7	1	2	2	2	1	2	2	2	2	0	0
pt4	D, C4	2	2	2	2	2	2	2	2	2	2	2
pt5	B, C7	2	2	2	2	2	2	2	2	2	2	2

2= innervated muscle, 1= partially denervated muscle, 0= denervated muscle.

AIS=American Spinal Cord Injury Impairment Scale, NLI=neurological level of injury

Supplementary file 2 Manual muscle test (MRC) results/ muscles included in MP integrity test

RIGHT MRC	ECU PRE	ECU POST	ECU DIF	ECR PRE	ECR POST	ECR DIF	EDC PRE	EDC POST	EDC DIF	EPL PRE	EPL POST	EPL DIF	APL PRE	APL POST	APL DIF	PRT PRE	PRT POST	PRT DIF	FDP PRE	FDP POST	FDP DIF	FPL PRE	FPL POST	FPL DIF	IOD 2-3 PRE	IOD 2-3 POST	IOD 2-3 DIF
pt 1	3	4	**1**	5	5	**0**	4	5	**1**	4	5	**1**	3	5	**2**	5	5	**0**	5	5	**0**	4	5	**1**	3	5	**2**
pt 2	5	5	**0**	5	5	**0**	4	5	**1**	5	5	**0**	5	5	**0**	5	5	**0**	4	5	**1**	5	5	**0**	4	5	**1**
pt 3	5	5	**0**	5	5	**0**	5	5	**0**	1	1	**0**	1	4	**3**	5	5	**0**	3	3	**0**	2	1	**-1**	0	0	**0**
pt 4	4	5	**1**	4	5	**1**	4	5	**1**	4	4	**0**	4	5	**1**	3	4	**1**	4	5	**1**	5	5	**0**	3	4	**1**
pt 5	5	5	**0**	5	5	**0**	3	3	**0**	1	4	**3**	0	1	**1**	5	5	**0**	1	3	**2**	1	4	**3**	2	4	**2**
LEFT MRC	ECU PRE	ECU POST	ECU DIF	ECR PRE	ECR POST	ECR DIF	EDC PRE	EDC POST	EDC DIF	EPL PRE	EPL POST	EPL DIF	APL PRE	APL POST	APL DIF	PRT PRE	PRT POST	PRT DIF	FDP PRE	FDP POST	FDP DIF	FPL PRE	FPL POST	FPL DIF	IOD 2-3 PRE	IOD 2-3 POST	IOD 2-3 DIF
pt 1	4	5	**1**	4	4	**0**	5	5	**0**	4	5	**1**	4	5	**1**	4	5	**1**	3	3	**0**	4	5	**1**	3	4	**1**
pt 2	5	5	**0**	5	5	**0**	4	4	**0**	5	5	**0**	5	5	**0**	5	5	**0**	5	5	**0**	5	5	**0**	4	5	**1**
pt 3	5	5	**0**	5	5	**0**	4	4	**0**	2	3	**1**	0	1	**1**	5	5	**0**	1	1	**0**	1	1	**0**	0	0	**0**
pt 4	4	4	**0**	4	4	**0**	3	4	**1**	4	4	**0**	4	4	**0**	4	4	**0**	4	4	**0**	4	4	**0**	3	4	**1**
pt 5	5	5	**0**	5	5	**0**	5	5	**0**	4	5	**1**	3	5	**2**	5	5	**0**	5	5	**0**	4	5	**1**	4	5	**1**

ECU=extensor carpi ulnaris, ECR=extensor carpi radialis, EDC=extensor digitorum communis, EPL=extensor pollicis longus, APL=abductor pollicis longus, PRT=pronator teres, FDP=flexor digitorum profundus, FPL=flexor pollicis longus, IOD2-3= interosseus 2-3 tested together, DIF=difference POST-PRE, MRC scale: 0= no contraction, 5= normal power

yellow= included in analysis

Supplementary file 3 List of muscles tested in MRC

**Muscle (additional)**	**Nerve**
serratus anterior	other
trapezius pars cranialis/ pars medialis	other
levator scapulae	other
rhomboideus minor & major	other
deltoideus pars ventralis/ pars lateralis/ pars posterior	other
latissimus dorsi	other
teres major	other
supraspinatus	other
pectoralis major	other
infraspinatus, teres minor	other
subscapularis	other
biceps brachii, brachialis, brachioradialis	other
**Muscle (stimulated)**	**Nerve**
triceps brachii	radial and axillary
supinator longus	radial
pronator teres, pronator quadratus	median
flexor carpi radialis	median
flexor carpi ulnaris	ulnar
extensor carpi radialis	radial
extensor carpi ulnaris	radial
flexor digitorum superficialis	median
flexor digitorum profundus I-II	median
flexor digitorum profundus IV-V	ulnar
extensor digitorum	radial
extensor digiti minimi	radial
extensor indicis	radial
interossei dorsalis	ulnar
abductor digiti minimi	ulnar
interossei palmares	ulnar
flexor pollicis brevis	median/ulnar
flexor pollicis longus	median
extensor pollicis brevis	radial
extensor pollicis longus	radial
abductor pollicis brevis	median
abductor pollicis longus	radial
adductor pollicis	ulnar
opponens pollicis	median
opponens digiti minimi	ulnar

Supplementary file 4 Motor point integrity testing points

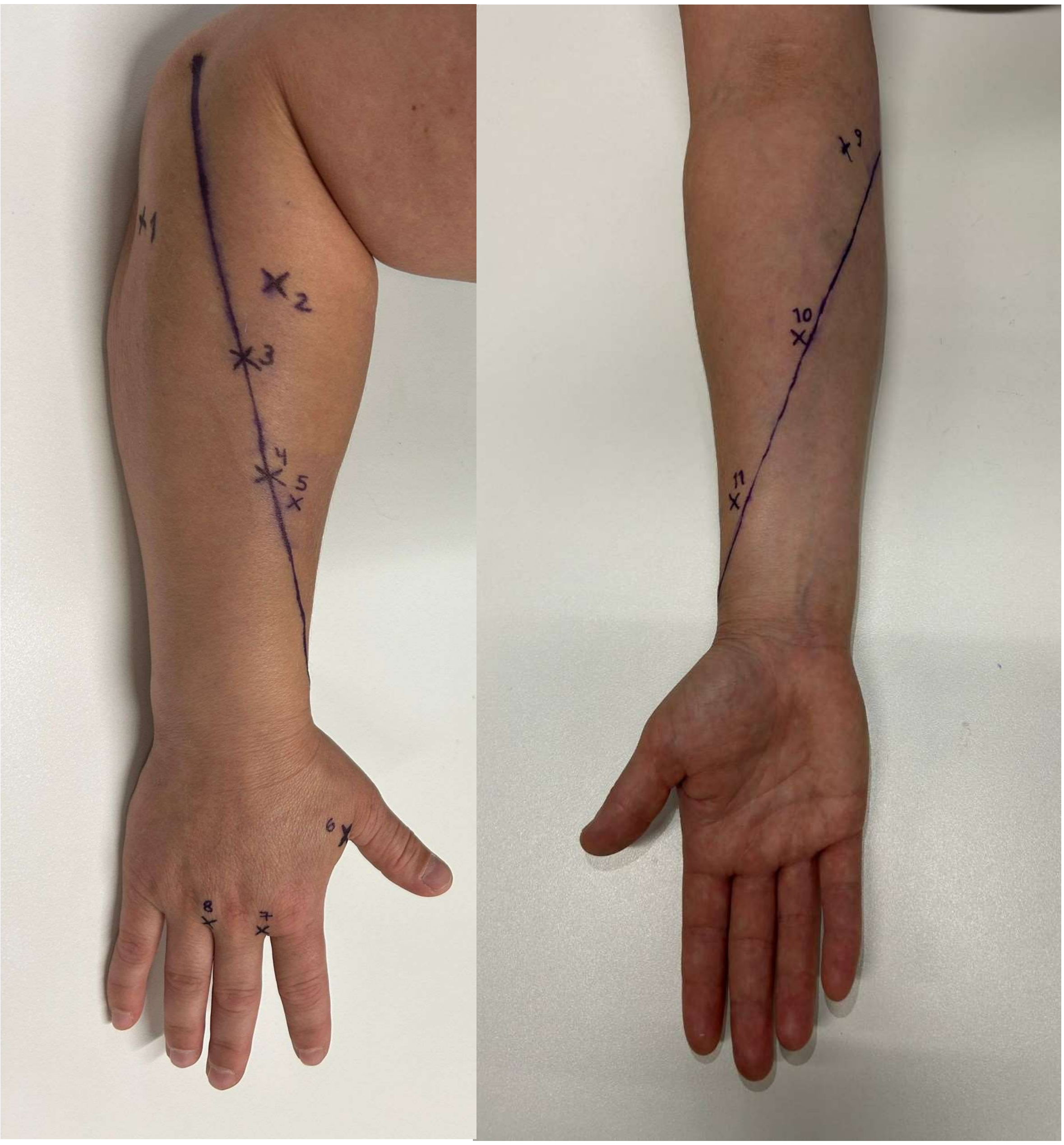

On the left: 1=Extensor carpi ulnaris, 2=Extensor carpi radialis, 3=Extensor digitorum communis, 4=Extensor pollicis longus, 5=Abductor pollicis longus, 6=Palmar interosseus 1, 7=Palmar interosseus 2, 8=Palmar interosseus 3. Landmark line from lateral condyle to radial styloid.

On the right: 9=Pronator teres, 10=Flexor digitorum profundus, 11=Flexor pollicis longus. Landmark line from medial condyle to radial styloid.

Supplementary file 4 Motor point integrity test cartography system

Charts for motor point integrity testing for clinical use. The illustration depicts a series of forearm lengths at a distance of 0.5 cm, accompanied by the calculation of the individual motor points.

MAPPING EXTENSOREN

Distanz	Faktor	Diagonale A/B	ECU	Abweichung nach ulnar	ECR	Abweichung nach radial	EDC	EPL	APL	Abweichung nach radial
	Baseline	25	4.5	3.7	7.5	1.7	10	14.7	15.5	0.50
1	0.92	23	4.14	3.40	6.90	1.56	9.20	13.52	14.26	0.46
2	0.94	23.5	4.23	3.48	7.05	1.60	9.40	13.82	14.57	0.47
3	0.96	24	4.32	3.55	7.20	1.63	9.60	14.11	14.88	0.48
4	0.98	24.5	4.41	3.63	7.35	1.67	9.80	14.41	15.19	0.49
5	1.00	25	4.50	3.70	7.50	1.70	10.00	14.70	15.50	0.50
6	1.02	25.5	4.59	3.77	7.65	1.73	10.20	14.99	15.81	0.51
7	1.04	26	4.68	3.85	7.80	1.77	10.40	15.29	16.12	0.52
8	1.06	26.5	4.77	3.92	7.95	1.80	10.60	15.58	16.43	0.53
9	1.08	27	4.86	4.00	8.10	1.84	10.80	15.88	16.74	0.54
10	1.10	27.5	4.95	4.07	8.25	1.87	11.00	16.17	17.05	0.55
11	1.12	28	5.04	4.14	8.40	1.90	11.20	16.46	17.36	0.56
12	1.14	28.5	5.13	4.22	8.55	1.94	11.40	16.76	17.67	0.57
13	1.16	29	5.22	4.29	8.70	1.97	11.60	17.05	17.98	0.58
14	1.18	29.5	5.31	4.37	8.85	2.01	11.80	17.35	18.29	0.59
15	1.20	30	5.40	4.44	9.00	2.04	12.00	17.64	18.60	0.60
16	1.22	30.5	5.49	4.51	9.15	2.07	12.20	17.93	18.91	0.61
17	1.24	31	5.58	4.59	9.30	2.11	12.40	18.23	19.22	0.62
18	1.26	31.5	5.67	4.66	9.45	2.14	12.60	18.52	19.53	0.63
19	1.28	32	5.76	4.74	9.60	2.18	12.80	18.82	19.84	0.64
20	1.30	32.5	5.85	4.81	9.75	2.21	13.00	19.11	20.15	0.65
21	1.32	33	5.94	4.88	9.90	2.24	13.20	19.40	20.46	0.66
22	1.34	33.5	6.03	4.96	10.05	2.28	13.40	19.70	20.77	0.67
23	1.36	34	6.12	5.03	10.20	2.31	13.60	19.99	21.08	0.68
24	1.38	34.5	6.21	5.11	10.35	2.35	13.80	20.29	21.39	0.69
25	1.40	35	6.30	5.18	10.50	2.38	14.00	20.58	21.70	0.70
26	1.42	35.5	6.39	5.25	10.65	2.41	14.20	20.87	22.01	0.71
27	1.44	36	6.48	5.33	10.80	2.45	14.40	21.17	22.32	0.72
28	1.46	36.5	6.57	5.40	10.95	2.48	14.60	21.46	22.63	0.73
29	1.48	37	6.66	5.48	11.10	2.52	14.80	21.76	22.94	0.74
30	1.50	37.5	6.75	5.55	11.25	2.55	15.00	22.05	23.25	0.75

FLEXOREN MAPPING

**Distanz**	**Faktor**	**Diagonale A/B**	**Pronator Teres**	Abweichung nach radial	**FDPIII**	Abweichung nach radial	**FPL**	Abweichung nach radial
	Baseline	25.5	6	2	15.5	0.5	21.5	0.8
1	0.90	23.0	5.41	1.80	13.98	0.45	19.39	0.72
2	0.92	23.5	5.53	1.84	14.28	0.46	19.81	0.74
3	0.94	24.0	5.65	1.88	14.59	0.47	20.24	0.75
4	0.96	24.5	5.76	1.92	14.89	0.48	20.66	0.77
5	0.98	25.0	5.88	1.96	15.20	0.49	21.08	0.78
6	1.00	25.5	6.00	2.00	15.50	0.50	21.50	0.80
7	1.02	26.0	6.12	2.04	15.80	0.51	21.92	0.82
8	1.04	26.5	6.24	2.08	16.11	0.52	22.34	0.83
9	1.06	27.0	6.35	2.12	16.41	0.53	22.76	0.85
10	1.08	27.5	6.47	2.16	16.72	0.54	23.19	0.86
11	1.10	28.0	6.59	2.20	17.02	0.55	23.61	0.88
12	1.12	28.5	6.71	2.24	17.32	0.56	24.03	0.89
13	1.14	29.0	6.82	2.27	17.63	0.57	24.45	0.91
14	1.16	29.5	6.94	2.31	17.93	0.58	24.87	0.93
15	1.18	30.0	7.06	2.35	18.24	0.59	25.29	0.94
16	1.20	30.5	7.18	2.39	18.54	0.60	25.72	0.96
17	1.22	31.0	7.29	2.43	18.84	0.61	26.14	0.97
18	1.24	31.5	7.41	2.47	19.15	0.62	26.56	0.99
19	1.25	32.0	7.53	2.51	19.45	0.63	26.98	1.00
20	1.27	32.5	7.65	2.55	19.75	0.64	27.40	1.02
21	1.29	33.0	7.76	2.59	20.06	0.65	27.82	1.04
22	1.31	33.5	7.88	2.63	20.36	0.66	28.25	1.05
23	1.33	34.0	8.00	2.67	20.67	0.67	28.67	1.07
24	1.35	34.5	8.12	2.71	20.97	0.68	29.09	1.08
25	1.37	35.0	8.24	2.75	21.27	0.69	29.51	1.10
26	1.39	35.5	8.35	2.78	21.58	0.70	29.93	1.11
27	1.41	36.0	8.47	2.82	21.88	0.71	30.35	1.13
28	1.43	36.5	8.59	2.86	22.19	0.72	30.77	1.15
29	1.45	37.0	8.71	2.90	22.49	0.73	31.20	1.16

Supplementary file 5 Outcome measures by patient and hand

Test	Baseline	Pre	1 month	2 months	3 months	4 months	End of PAS	Follow-up 1 mo	Follow-up 3 mo	Follow-up 6 mo	Follow-up 12 mo
**MRC mean stimulated**											
pt 1	3,45	3,91	4,55	4,64	4,73	4,82	4,32	4,23	4,32	4,64	
pt 2	4,05	3,95	4,63	4,89			4,79	4,68	4,74	4,68	4,53
pt 3	2,18	1,76	2,35	2,47	2,47		2,59	2,53	2,59	2,71	2,59
pt 4	3,46	3,57	3,75	4,04	4,33		4,54	4,63	4,63	4,71	4,67
**Box and Block stimulated**											
pt 1	42	43	43	49	48	48	50	50	53	49	
pt 2	51	44	48	50			51	48	48	47	45
pt 3	56	61	58	59	65		61	61	59	63	62
pt 4	35	38	45	51	46		47	55	55	49	46
**9-Hole Peg stimulated**											
pt 1	7	6	9	7	17	8	8	14	18	14	
pt 2	18	17	18	18			18	18	18	18	8
pt 3	18	18	18	18	18		18	18	18	18	18
pt 4	16	18	18	18	18		15	18	18	18	18
**Hand dynamometry stimulated**											
pt 1	30	29	30	34	34,5	40	32	36	34	35	
pt 2	18	20	20	18			19	20	18	16	17
pt 3	0	0	0	0	0		0	0	0	0	0
pt 4	12	16	16	27	28		28	30	26	27	27
**Tip pinch stimulated**											
pt 1	7	5,25	5	6,25	7,75	8,5	6	8	6,5	7,5	
pt 2	5,75	5,75	4,5	6			4	5,25	4	3,75	4,5
pt 3	0	0	0	0	0,5		0	0,25	0	0	0
pt 4	4	5	4,5	6	6,5		5,5	6,5	5,25	7	7
**Key pinch stimulated**											
pt 1	8,5	9,75	9,25	9,5	11	11	9,75	10,5	11	10,5	
pt 2	5,75	6	6,5	7,5			6	6,75	6,5	5	5,75
pt 3	0	0,5	0,5	0,5	0,5		0,5	0,5	0,5	0,5	0,5
pt 4	6,5	6	6	8	8		7	8	7,5	9	8
**Palmar pinch stimulated**											
pt 1	8	6,5	8	8,25	10	8,75	9,25	9,75	9,5	9,75	
pt 2	5	4,5	4,5	3,5			4,5	4,5	4	3,5	3,5
pt 3	0	0	0	0	0,5		0	0,5	0	0	0,25
pt 4	6	5,5	5,25	6,75	8,25		7	8,5	8,5	8	7,5
**ASIA motor sum stimulated**											
pt 1	22	24	25	25	25	25	25	24	25	25	
pt 2	24	24	25	25			25	25	25	25	23
pt 3	23	19	20	21	21		22	22	22	22	22
pt 4	17	17	17	23	24		24	24	24	24	24
**ASIA LT sum stimulated**											
pt 1	10	13	16	18	18	15	18	15	16	15	
pt 2	17	15	16	18			17	16	18	18	16
pt 3	17	18	18	17	17		16	15	15	16	16
pt 4	14	15	16	16	16		16	16	15	16	15
**ASIA PP sum stimulated**											
pt 1	11	14	17	12	18	10	16	13	16	11	
pt 2	16	14	18	14			18	18	18	18	14
pt 3	12	14	15	15	14		13	13	14	15	15
pt 4	8	4	8	9	11		12	13	11	8	9
**MAS sum stimulated**											
pt 1	3	4,5	4,5	3	5	4	4	5	2	6	
pt 2	3	6	2	3			3	4	5	5	7
pt 3	0	0	0	0	0		0	0	0	0	0
pt 4	5	4,5	3	3,5	1		1	2	1	3	2
**SCIM total**											
pt 1	68	68	68	68	68	68	68	76	76	75	
pt 2	89	87	87	87			89	90	90	90	90
pt 3	61	59	59	60	60		61	61	61	61	61
pt 4	75	75	82	84	86		86	86	86	86	86

Test	Baseline	Pre	1 month	2 months	3 months	4 months	End of PAS	Follow-up 1 mo	Follow-up 3 mo	Follow-up 6 mo	Follow-up 12 mo
**MRC mean unstimulated**											
pt 1	3,57	3,91	4,57	4,70	4,57	4,78	4,70	4,48	4,57	4,57	
pt 2	3,80	4,10	4,70	4,90			4,90	4,70	4,70	4,60	4,40
pt 3	1,33	1,53	2,00	1,89	2,00		2,06	2,00	2,06	1,94	2,00
pt 4	3,76	3,56	3,80	4,04	4,04		4,08	4,20	4,16	4,48	4,44
**Box and Block unstimulated**											
pt 1	44	42	44	49	46	44	47	44	50	46	
pt 2	42	43	43	43			46	42	41	46	43
pt 3	46	50	51	47	51		53	57	51	50	48
pt 4	38	44	48	51	52		49	58	55	53	54
**9-Hole Peg unstimulated**											
pt 1	18	18	14	18	18	15	14	18	11	12	
pt 2	9	10	14	11			8	11	16	7	5
pt 3	18	12	18	3	18		6	18	10	18	7
pt 4	18	18	18	18	18		18	18	18	18	18
**Hand dynamometry unstimulated**											
pt 1	24	27	30	28	32	34	35	30	37	36	
pt 2	23	24	24	25			22	20	20	20	21
pt 3	0	0	0	0	0		0	0	0	0	0
pt 4	20	15	14	24	27		24	25	26	26	22
**Tip pinch unstimulated**											
pt 1	3,75	5,25	7	4,75	6,5	8	7,5	7,5	7,5	7	
pt 2	4,75	5	5	5,5			4	5,25	3	5,5	5,25
pt 3	0	0	0	0	0		0	0	0	0	0
pt 4	5	5	5,25	7	8		7	7	7	6	6,5
**Key pinch unstimulated**											
pt 1	8	9,5	8,5	8,5	10	10,5	8,5	10,5	9,5	9	
pt 2	7	7	6,5	6,5			6	6	4,5	6	5
pt 3	0	0	0	0	0		0	0	0,5	0,5	0,25
pt 4	7	7	7	7,5	8		8	8,25	7,75	8	7,5
**Palmar pinch unstimulated**											
pt 1	4,75	6,5	7,5	6,25	9	8	10	9,5	8,5	8,75	
pt 2	6	5,25	5,75	5,75			5,25	6	4,5	5	4,75
pt 3	0	0	0	0	0		0	0	0	0	0
pt 4	6	5	5,25	8,5	9,5		7,5	9,25	8,75	8	7,5
**ASIA motor sum unstimulated**											
pt 1	24	23	25	25	25	25	25	25	25	24	
pt 2	25	24	25	25			25	25	25	25	25
pt 3	22	16	16	16	16	16	16	16	16	16	
pt 4	18	20	20	20	21		21	21	21	24	22
**ASIA LT sum unstimulated**											
pt 1	14	14	15	16	15	17	16	16	17	16	
pt 2	15	16	17	15			17	17	18	18	18
pt 3	17	18	15	15	17		16	15	15	15	16
pt 4	13	14	14	16	17		16	16	14	16	15
**ASIA PP sum unstimulated**											
pt 1	18	16	18	18	17	18	18	18	18	18	
pt 2	18	17	17	15			18	11	16	18	13
pt 3	12	13	12	12	12		14	13	15	13	14
pt 4	9	8	10	13	14		15	14	13	12	11
**MAS sum unstimulated**											
pt 1	3	1	1	4	3	1	2	2	1	4	
pt 2	0	1	0	0			0	1	2	1	2
pt 3	0	0	1	1	1		1	1	1	0	1
pt 4	5,5	3	2	1	2		0	0	0	1	1
